# Indoor Environmental Determinants of Depression: A New Approach to Understanding Mental Health

**DOI:** 10.3390/medicina62030496

**Published:** 2026-03-06

**Authors:** Gintare Kaliniene, Ruta Ustinaviciene, Rasa Zutautiene, Jolita Kirvaitiene, Abdonas Tamosiunas, Vaiva Lesauskaite, Dalia Luksiene

**Affiliations:** 1 Department of Environmental and Occupational Medicine, Public Health Faculty, Lithuanian University of Health Sciences, Tilžės St. 18, 47181 Kaunas, Lithuania; gintare.kaliniene@lsmu.lt (G.K.); ruta.ustinaviciene@lsmu.lt (R.U.); rasa.zutautiene@lsmu.lt (R.Z.); jolita.kirvaitiene@lsmu.lt (J.K.); 2 Health Research Institute, Lithuanian University of Health Sciences, Tilžės St. 18, 47181 Kaunas, Lithuania; 3 Laboratory of Population Studies, Institute of Cardiology, Medical Academy, Lithuanian University of Health Sciences, Sūkilėlių Ave. 15, 50103 Kaunas, Lithuania; abdonas.tamosiunas@lsmu.lt; 4 Laboratory of Molecular Cardiology, Institute of Cardiology, Medical Academy, Lithuanian University of Health Sciences, Sukilėlių Ave. 15, 50103 Kaunas, Lithuania; vaiva.lesauskaite@lsmu.lt

**Keywords:** depressive symptoms, indoor environment, mold, odors, room ventilation

## Abstract

*Background and Objectives:* Depression has emerged in recent years as a significant global health issue, drawing considerable research interest and attention. The development of depression could be impacted by a range of environmental factors. Aim: To investigate the relationship between depressive symptoms and various indoor environmental factors, such as microclimate, odors, mold, and room ventilation, in association with some sociodemographic and lifestyle factors. *Materials and Methods:* This epidemiological health survey of the study “Chronic diseases and their risk factors in the adult population” was performed during 2023–2024 in Kaunas city (Lithuania) following the methodology of the WHO MONICA study. A random sample of Kaunas inhabitants aged 25–69 years, stratified by sex and age, was randomly selected from the Lithuanian population register. The 3426 individuals were screened. The associations of various indoor environmental factors with depressive symptoms were investigated using binary logistic regression analysis. *Results:* Depressive symptoms were associated with sociodemographic, lifestyle, and indoor environmental factors. Poor microclimate conditions, unpleasant household odors, mold exposure, and insufficient room ventilation were associated with increased odds of depressive symptoms. The significance of these associations varied across sex, age, marital status, socioeconomic status, and physical activity of responders. Additional multivariable logistic regression analyses, including interaction terms between each indoor environmental factor and the stratification variables (sex, age groups, marital status, family economic situation, and physical activity), were performed. Significant interaction was found only between family status and room ventilation (*p* = 0.007). This indicates that the association between ventilation and depressive symptoms differed by family status. *Conclusions:* This study contributes to the cross-disciplinary understanding of the role of indoor environmental quality, sociodemographic, and lifestyle factors in the development of depression, adding to the evidence on the role of other factors in depression inequalities.

## 1. Introduction

Depression, classified as a non-communicable disease, has emerged in recent years as a significant global health issue, drawing considerable research interest and attention [1,2]. According to World Health Organization data, approximately 332 million people in the world suffer from depressive disorders. Depression is about 1.5 times more common among women than among men [3]. Furthermore, the prevalence of depression exhibits a peak during middle and late adulthood, affecting approximately 11.5% to 13.5% of individuals aged 65 years and older [4]. In Lithuania, the proportion of the population experiencing depressive symptoms of varying severity increases with age. Among individuals aged 55–64, up to 22% report such symptoms, while among those aged 75 and older, the prevalence reaches as high as 35% [5]. These individual-level risk characteristics for depression are highly important, but it is also essential to study environmental risk factors for this major public health concern [6].

Depression development is impacted by a range of environmental factors—among them increased ambient air pollution [7,8], outdoor and indoor noise [9,10,11,12], and geomagnetic storms [13]. Despite the fact that publications more often analyze the lack of green spaces, noise, and air pollution, which were clearly associated with depressive mood, attention should be paid to less frequently analyzed factors, such as microclimate, odors, mold, and room ventilation [6]. The indoor environment of a building, such as temperature, humidity, and air quality, affects physical comfort, such as touch, hearing, smell, and sight, which in turn affects a person’s mental health [1]. Therefore, in this study, we focus on the associations between depressive symptoms and specific factors of the indoor environment.

Recently, there has been growing evidence that indoor cold and heat exposures are associated with adverse health effects. Highlighting the health risks associated with abnormal indoor temperatures [14]. Whereas high indoor temperatures have been associated with sleep disturbance, increased hospital admissions, and higher mortality rates. Low indoor temperatures are associated with increased risks of cardiovascular and respiratory diseases, poor sleep quality, reduced physical performance, and poor self-rated health. Regarding mental and general health, perceived cold (which is a subjective measure) has been reported to increase risks of psychological distress and affect quality of life [15,16].

In addition, multiple studies have confirmed that depression and anxiety are also associated with environmental factors, including exposure to outdoor and indoor air pollution. Existing evidence suggests that exposure to indoor musty odors constitutes a chronic psychological stressor, potentially contributing to the onset and worsening of mental health problems [17]. Musty odors are a common problem in indoor environments and are caused by the presence of mold and dampness. The results revealed a significant association of indoor musty odors with depression and anxiety symptoms [18], and with cognitive impairment among older adults [19].

Another environmental factor that constantly draws the attention of researchers is indoor mold. According to a large review conducted by Australian researchers, the available evidence indicates positive associations between residential dampness or mold exposure and poor mental health outcomes. In adults, these associations were observed for depression, stress, and anxiety, while in children, they were noted for emotional symptoms and emotional dysregulation [18]. Chinese researchers found a prevalence of 13.61% for depression and 11.79% for anxiety. Participants exposed to mold had significantly higher odds ratios of depression and anxiety compared to participants not exposed to mold [20].

And the last factor of the living environment that we will discuss in this study is the ventilation of the indoor environment and its associations with depression. Worldwide, people spend roughly 80–90% of their time in indoor environments [21]. It was determined that infrequent indoor ventilation is linked to higher levels of depressive symptoms among older adults. It may be related to the fact that ventilation can effectively improve indoor air quality, increase indoor oxygen content, and remove indoor harmful gases and transmission, while air quality is negatively correlated with depressive symptoms [22]. Scientific articles typically examine living environmental factors separately—for example, indoor temperature, humidity, odors, or mold—and analyze their individual associations with depressive symptoms. In contrast, the present study incorporates all the mentioned living environment factors (including microclimate, odors, mold, and room ventilation) and evaluates their associations with depressive symptoms. Therefore, this study aimed to investigate the relationship between depressive symptoms and various indoor environmental factors, such as microclimate, odors, mold, and room ventilation, in association with some sociodemographic and lifestyle factors.

## 2. Materials and Methods

### 2.1. Study Population

This epidemiological health survey of the study “Chronic Diseases and their Risk Factors in the Adult Population” (Study) was performed in Kaunas city (Lithuania) following the methodology of the WHO programme Multinational Monitoring of Trends and Determinants in Cardiovascular Disease (MONICA) study [23]. A random sample of Kaunas males and females aged 25–69 years, stratified by sex and age, was randomly selected from the Lithuanian population register. Since about 50–60% of the sample usually agrees to participate in epidemiological research of this type, it was planned to form a random sample of 6000 individuals (3000 men and 3000 women, 750 from each decade of age). A random sample of subjects stratified by age group and sex (N = 6000) was drawn from the lists of residents of Kaunas city. This sample was drawn by specialists from the Lithuanian Population Register. Invitations were sent to the selected individuals to attend a health check-up at the Hospital of the Lithuanian University of Health Sciences, Kaunas Clinics. The call for participants started on 1 February 2020, but due to the COVID-19 pandemic, the Study was suspended on 15 March 2020, and in March 2023, the study was resumed and was conducted until 21 June 2024. The 3426 individuals (1523 males and 1903 females) were screened. The response rate was 57.1%. A total of 3175 participants (1791 women and 1384 men) were available for statistical analysis after excluding 251 responders with missing information on study variables.

### 2.2. Variables Determined Using the Questionnaire

During the health examination, trained interviewers gathered sociodemographic and health-related data using a structured questionnaire, which included items on age, sex, marital status, and educational level. Education was measured by six education levels: primary, incomplete secondary, secondary, vocational, college, and university. Finally, education level was grouped into three groups: first, the responders with primary, incomplete secondary, and vocational education were considered as having secondary and lower education; second, responders with college education level, and third group responders with university education level. Marital status was dichotomized as married (married, cohabiting) and single (single, divorced, separated, and widowed).

For assessing smoking habits, respondents were asked: “Do you smoke at the moment?” Answers: 1—yes, daily at least one cigarette a day on average; 2—former (No, I smoked in the past, but I stopped), 3—no, I have never smoked. Smoking was categorized into two groups: never/former, and current regular smoking (smoking at least 1 cigarette per day each day). To assess physical activity during leisure time, respondents were asked: “How many hours per week during your leisure time do you spend separately in the autumn-winter and spring-summer seasons? Answers (in hours) for: walking, gardening, house maintenance, and other physical activities. Physical activity was calculated by summarizing time spent per week separately in the autumn-winter and spring-summer seasons for activities such as walking, gardening, house maintenance, and other physical activities. The participants were divided into three equal groups (tertiles) according to the mean length of time spent per week on physical activities. The 1st tertile maximal cut-off was 5 h per week (mean 2.83 (standard deviation (SD) = 1.53)), the 2nd tertile maximal cut-off was 9.5 h per week (mean 7.25 (SD = 1.24)), and the 3rd tertile maximal cut-off was 42 h per week (mean 15.3 (SD = 5.85)).

Depressive symptoms were evaluated using the 10-item Center for Epidemiologic Studies Depression Scale, (CES-D 10) [24,25]. All the participants were asked to answer 10 questions to evaluate the presence of depressive symptoms during the past week. Each answer was rated as either 0 or 1, 0 meaning “no” and 1 meaning “yes”. The maximal possible sum of the evaluation points ranged from 0 to 10. If the sum of the scores was equal to or greater than 4, responders were regarded as having depressive symptoms [26].

The family’s economic situation was evaluated according to the answer of the health survey participants to the question “According to your family’s monthly income, how would you describe your family’s economic situation in one statement?” Possible response: very good, good, poor. Membership of a club/organization was evaluated according to the answer “Are you a member of a club or organization (e.g., sports club, church, political party)? Possible response: Yes/No.

#### Indoor Environmental Factors Evaluation

To investigate the relationship between depressive symptoms and various indoor environmental factors, including residential microclimate conditions and perceived odors, an assessment was conducted using the Indoor Air Quality–Preliminary Occupant Questionnaire [27]. This instrument is widely used in environmental and occupational health investigations as a preliminary screening tool for the subjective experience of indoor environmental quality [28]. According to Bluyssen (2016), subjective indicators are essential for understanding real occupant discomfort, as physical measurements alone cannot fully explain how people experience the indoor environment [29].

The microclimate score was evaluated using the 6-item scale [27]. The microclimate score is the sum of the answer options for six questions about the microclimate of the indoor environment. The answer options for each of the six questions range from 1 (never) to 5 (every day). The minimum sum of the microclimate score is 6, the maximum is 30. First-good microclimate, the score is lower than the median (median < 7); second-poor microclimate, the score is higher than the median (median ≥ 7).

The odor score was evaluated using the 5-items scale [27]. The odor score is the sum of the answer options for five questions about odors in the indoor environment. The answer options for each of the five questions range from 1 (never) to 5 (every day). The minimum sum of the odor score is 5, the maximum is 25. The median of the odor score is 6. The odor status was divided into two groups: 1st—no odors, the score is lower than the median (median < 6); 2nd—yes odors, the score is higher than the median (median ≥ 6).

Mold was evaluated according to the answer, “ Is there mold in your indoor environment?” Possible response: Yes/No. If responders answered “Yes”, they were additionally asked to indicate in which living environment the mold is Possible response: in indoor rooms, around windows, on the balcony, in the bathroom.

Room ventilation was evaluated according to the answer “Do you ventilate your indoor spaces daily?” Possible response: Yes/No.

### 2.3. Measurement

The body weight was measured with a calibrated medical scale, without shoes or heavy clothes. Weight values were recorded to the nearest 100 g. The height of participants (without shoes) was measured with an accuracy of one centimeter, using a stadiometer. Body mass index (BMI) was calculated using the following formula: BMI = body mass (kg)/height^2^ (m^2^). The health survey participants were divided into groups: group with normal weight (BMI < 24.99 kg/m^2^), overweight (BMI 25.0–29.99 kg/m^2^), and obese (BMI ≥ 30.0 kg/m^2^).

### 2.4. Statistical Analysis

All statistical analyses in this investigation were executed using IBM SPSS Statistics (Version 29.0) (IBM Corp. Released 2023. IBM SPSS Statistics for Windows, Version 29.0. Armonk, NY, USA). Baseline characteristics for continuous variables were described as mean (standard deviation (SD)), and categorical variables were denoted as frequency (n) and relative frequency (%). For the evaluation of the association of microclimate and odor with depressive symptoms, we used continuous and categorical values. We tested whether the microclimate scores and odor scores were normally distributed using the Shapiro–Wilk test. Since neither the microclimate nor the odor scores were normally distributed, we used the median when dividing into two groups: less than the median and equal to or greater than the median.

The differences in distribution of responders of the Kaunas health survey (2023–2024) with depressive symptoms according to baseline characteristics were evaluated using a chi-squared and Z test with Bonferroni corrections. *p* < 0.05 values were considered statistically significant for assessing the differences in categorical variables.

The associations of sociodemographic, lifestyle factors, family’s economic situation, membership of a club/organization, and indoor environmental factors with depressive symptoms were investigated using binary logistic regression analysis. The odds ratios (OR) with 95% confidence interval (CI) were computed. Two models were used: Model 1: simple binary logistic regression for each independent variable; the dependent variable being depressive symptoms. For a multivariable binary logistic regression (Model 2) analysis of the associations between sociodemographic, lifestyle factors, family economic situation, membership in a club/organization, indoor environmental factors, and depressive symptoms, the dependent variable was depressive symptoms. Independent variables: sex, age, education, marital status, lifestyle factors (smoking status, BMI, physical activity status in leisure time), family’s economic situation, membership of a club/organization, indoor environmental factors (microclimate, odors, mold, room ventilation daily).

For multivariable binary logistic regression analysis of associations of indoor environmental factors with depressive symptoms in different sexes, ages, family’s economic situation, marital status, and physical activity groups, the dependent variable was depressive symptoms. Independent variables: indoor environmental factors (microclimate, odors, mold, room ventilation daily). Covariates: sex, age, education, marital status, lifestyle factors (smoking status, body mass index, physical activity status in leisure time), family’s economic situation, membership of a club/organization.

### 2.5. Ethical Approval

The Study was approved by the Kaunas Regional Ethics Committee (Lithuania) (Nr. BE-2-49; 5 June 2018). All participants provided written informed consent. Inclusion criteria: all selected people are invited. No exclusion criteria were applied.

## 3. Results

### 3.1. Baseline Characteristics of Responders

The baseline characteristics of responders are presented in Table 1. A total number of participants included in the Kaunas health survey (2023–2024) was 3175. The participants’ average age was 49.4 ± 11.12 years, and 43.6% were male. The distribution according to education level revealed a high prevalence of a university degree (49.8%), 31.4% of participants had completed secondary education, and 18.8% had a college degree. More than two-thirds of participants were defined as married or in a partnership (72.8%), and 27.2% were single, divorced, or widowed.

Behavioral indicators analysis revealed quite a high prevalence of obese or overweight respondents (26.2% and 37.6%, respectively). The mean BMI was 27.3 ± 5.27 kg/m^2^ in the study population. The median weekly physical activity during leisure time was 7 h. Smokers accounted for 19.5% of the sample. The prevalence of the outcome variable-depressive symptoms (CES-D-10 ≥ 4) was 18.6%. Self-rated family’s economic situation was considered as very good (67.3%) or good (26.8%), while 5.9% reported it as poor. Additionally, 21.2% of participants were members of a club or organization.

The study’s independent variables–factors of indoor environment (microclimate, odors, mold, room ventilation) were evaluated. Their frequency analysis revealed that the median microclimate and odor ratings were 7 and 6, respectively. Mold was reported by 11.4% of respondents, and 94.1% reported ventilating their rooms daily.

### 3.2. Distribution of Depressive Symptoms According to Baseline Characteristics of Responders

The distribution of respondents with and without depressive symptoms according to sociodemographic, lifestyle, and environmental factors is provided in Table 2. The prevalence of depressive symptoms (CES-D-10 ≥ 4) was significantly higher among females, compared with the male group, among participants of the youngest age group, among single, divorced, or widowed participants, compared to married or in partnership, among those with normal BMI, with the lowest physical activity level, among those who rated their economic situation as poor (*p* < 0.001). No significant associations were found between depressive symptoms and education level, smoking status (in both female and male groups), and membership of club/organization (*p* > 0.05). Distribution analysis of depressive symptoms and indoor environmental factors revealed a significant association in all observed environmental factors. The respondents who reported a poorer microclimate or odor exposure had a significantly higher prevalence of depressive symptoms (in both cases, *p* < 0.001). Additionally, depressive symptoms were more prevalent among respondents who did not ventilate their rooms daily (*p* = 0.021), as well as mold exposure, which was also associated with a higher prevalence of depressive symptoms (*p* < 0.001).

### 3.3. Association of Depressive Symptoms with Sociodemographic, Lifestyle, and Indoor Environment Factors

The odds ratios for depressive symptoms according to independent variables are presented in Table 3. Firstly, a simple binary logistic regression analysis (Model 1) was performed. Secondly, in order to evaluate the association of sociodemographic, lifestyle, and indoor environment factors with depressive symptoms, a fully adjusted Model 2 was performed using multivariable binary logistic regression analysis.

In Model 1, the OR of depressive symptoms for female sex was significantly higher compared to males (OR = 1.45, 95% CI: 1.21–1.75, *p* < 0.001). Also, those who were single, divorced, or widowed had a significantly higher OR of depressive symptoms compared with those who were married or in a partnership (OR = 1.87, 95% CI: 1.55–2.26, *p* < 0.001). The OR of depressive symptoms was significantly associated with age. Being overweight or obese had a protective effect for depressive symptoms compared to those with a normal BMI ORs, respectively, were OR = 0.72, 95% CI: 0.59–0.86, *p* = 0.002; and OR = 0.70, 95% CI: 0.56–0.88, *p* = 0.003. Self-rated family’s economic situation was significantly associated with depressive symptoms. Those who considered themselves poor economic situation had more than threefold higher odds of depressive symptoms compared to those reporting a very good economic situation (OR = 3.71, 95% CI: 2.72–5.07, *p* < 0.001).

To evaluate the association between microclimate, odor, and depressive symptoms, we used both categorical and continuous variables. The multivariable logistic regression analysis showed that each one-point increase in microclimate and odor scores was associated with higher odds of depressive symptoms, by 13% (OR = 1.13; 95% CI: 1.09–1.17; *p* < 0.001) and by 4% (OR = 1.04; 95% CI: 1.01–1.07; *p* = 0.004), respectively. When categorical variables based on the median were used, poor microclimate and poor odor conditions were significantly associated with depressive symptoms (respectively OR = 1.40; 95% CI: 1.14–1.71; (*p* = 0.001), and OR = 1.45; 95% CI: 1.18–1.77; (*p* < 0.001)) (Table 3, Model 2).

For the evaluation of the association between mold presence and depressive symptoms, we used categorical variables. The multivariable logistic regression analysis showed that mold presence was significantly associated with depressive symptoms (OR = 1.53; 95% CI: 1.17–2.01; *p* = 0.002) (Table 3, Model 2).

According to results presented in Table 3, we analyzed the associations of indoor environmental factors with depressive symptoms in different sexes, ages, family’s economic situation, marital status, and physical activity responder groups. At first, additional multivariable logistic regression analyses, including interaction terms between each indoor environmental factor and the stratification variables (sex, age groups, marital status, family economic situation, and physical activity), were performed. Significant interaction was found only between family status and room ventilation (*p* = 0.007) (Table 4). This indicates that the association between ventilation and depressive symptoms differed by family status.

Associations of indoor environmental factors with depressive symptoms in different sexes, ages, family’s economic situation, marital status, and physical activity responder groups are presented as stratified analyses for descriptive purposes in Table 4.

The results show in the men group, only the presence of household odors was significantly associated with higher odds of depressive symptoms. In the women’s group, poor living environmental conditions (poor microclimate, odors, and mold exposure) were statistically significantly associated with depressive symptoms. After evaluating the associations between indoor environmental factors and depressive symptoms, in the group of family’s economic situation “very good + good”, poor living environmental conditions (poor microclimate, odors, and mold) were statistically significantly associated with increased odds of depressive symptoms. In a group of responders with a poor family economic situation, only the poor microclimate demonstrated significant associations with depressive symptoms. After evaluating the associations between indoor environmental factors and depressive symptoms in different responders’ marital status groups, the results show that in group “Married or cohabiting”, poor living environmental conditions (poor microclimate, odors, mold, and no daily room ventilation) showed statistically significant associations with increased odds of depressive symptoms. In the group of responders “single, divorced, widowed”, only poor microclimate and odors were associated with increased odds of depressive symptoms. The results show the associations between indoor environmental factors and depressive symptoms in the group 25–34 years, only the presence of household odors was significantly associated with a higher odd of depressive symptoms. In group 35–44 years, only the presence of mold was significantly associated with a higher odd of depressive symptoms. In the group 55+ years, the poor microclimate and presence of household odors were significantly associated with a depressive symptoms. In the physically active group (2 + 3 tertile), poor living environmental conditions (odors, mold, no daily room ventilation) were associated with increased odds of depressive symptoms. In the physically inactive group (1 tertile) of responders, only poor microclimate and odors were associated depressive symptoms.

In summary, poorer indoor environmental quality was associated with higher odds of depressive symptoms, and these associations varied across sociodemographic and lifestyle characteristics. Further longitudinal research is needed to clarify the direction and underlying mechanisms of these relationships.

## 4. Discussion

Depression is one of the main determinants of quality of life and causes of mortality in individuals of all ages. Many studies reveal the associations of individual factors with the development of depression, but there is controversial evidence about the relationship of socio-economic status, living environment, and lifestyle with depression [6,15,20]. We aimed to determine whether the living environment, its condition, mediates the relationship between lifestyle and socioeconomic status and depression, and what the interactive or common relationships are between these factors and their associations with depressive symptoms.

Despite the fact that people invest a lot of financial, creative, and emotional resources in their homes, research on housing and mental health is underdeveloped. Living conditions are widely recognized as important social health factors that determine mental health [5,6,22]. We examined the main subjective components and their relationship with depressive symptoms. One of our main findings was that residential odors and mold increased the odds ratio of depressive symptoms. The damage caused by moisture and its byproduct, mold, is multifaceted. Mold releases many components whose health effects cannot be assessed as individual substances [18,30]. Therefore, most epidemiological studies only assess the presence of mold in the environment, without examining air quality. This is confirmed by the analysis of many studies conducted. Molds in residential areas release metabolites that are either broad-spectrum antibiotics or cytotoxic mycotoxins. These substances affect residents, causing adverse health symptoms such as neuroimmunological and mental health effects [18,20,30]. Depression, psychological stress, tissue damage, malignancy, carcinogenesis, chronic fatigue syndrome, and experimental allergic encephalomyelitis can be induced at very low physiological concentrations by mycotoxin-induced natural killer cell activation in the brain. Ambient odors can affect our well-being in many ways. They can be an irritant, a factor that disrupts relaxation, or, in rare cases, cause nausea and headaches [19,30]. So, long-term exposure to toxigenic molds can lead to various neurological disorders, including headaches, general aches, fever, cough, memory impairment, depression, mood swings, sleep problems, anxiety, chronic fatigue, and seizures. These disorders can be considered precursors of somatic dysfunctions and are related to the development of diseases. Mold exposure also acts on the immune system and influences abnormal natural killer cell activity, which later can relate to depression, sleep disturbances, and other neurological disorders [31]. Summarizing, there is direct evidence that exposure to mold in the home is associated with measurable changes in inflammatory biomarkers in humans: Beijer et al. found that individuals living in homes with higher levels of airborne mold had more inflammatory cytokines, released from immune cells, compared to those living in homes with low levels of mold. This study suggests increased inflammatory responses associated with mold exposure [32]. Numerous publications on the neurobiology of inflammation in depression document how inflammatory signaling is associated with neurobiological systems implicated in depression: cytokines affect neurotransmitter systems and neuroplasticity-chronic inflammation can alter monoamine (e.g., serotonin, dopamine) levels, reduce neurotrophic factors, and disrupt glutamate signalling, all of which are associated with depressive symptoms [33,34]. These changes are manifested by cortisol release and altered stress responses, which are common features of depressive disorders. These mechanisms are central to models that suggest that immune activation can alter neurotransmission and neural circuitry.

As we can see from our data, poor microclimate, the presence of mold in the environment, and unpleasant odors were associated with depressive symptoms. This was confirmed by many studies and the previously discussed mechanisms of influence on behavior [17,30]. However, we aim to look deeper into this issue and clarify the personal and socioeconomic connections associated with the home environment and depressive symptoms. Therefore, it might be useful to combine exposures into common combined measures when used to assess the association with health.

Smell is one of the oldest and most fundamental senses, informing us about the environment and its threats [17]. Throughout human history, olfactory experience has been directly related to the internal environment, and therefore unpleasant odors are stressors that can trigger unpleasant memories and past negative emotions [35,36]. Therefore, it is necessary to more closely monitor the associations of home odors with people’s mental health. Thus, in our opinion, poor microclimate, home odors, and mold were the environmental factors most consistently associated with depressive symptoms, but sociodemographic and lifestyle factors can moderate the associations.

In our study, in the women’s group, several adverse environmental conditions were found as significant variables for depressive symptoms. In many studies, women have more complaints and evaluate their indoor environment more unfavorably. This may be due to the fact that they are more sensitive to the environment, spend more time at home, and pay more attention to living conditions, as they also care about the environment of their family members [37].

In most studies, a lower socioeconomic class is more likely to be associated with depression. This is shown by many publications [1,17,18,22,37]. In our study self-rated family’s economic situation was significantly associated with depressive symptoms. In the group family’s economic situation “very good + good”, poor living environmental conditions (poor microclimate, odors, and mold) demonstrated statistically significant associations with depressive symptoms. In a group of responders with a poor family economic situation, only the poor microclimate demonstrated significant associations with increased odds of depressive symptoms. This can be explained by several aspects. As publications show, when considering the causes and psychological mechanisms of depression, we find that an individual’s perception of their environment and psychosocial appraisals are associated with depression, which helps to explain our findings. For residents, lack of control over their home environment is a mediating factor in the association between exposure to unpleasant odors or mold and depressive symptoms. Residents who feel they have no control over their housing conditions and who hold negative perceptions of their environment—particularly when it falls short of their expectations–report higher levels of depressive symptoms [38,39]. In Lithuania, the vast majority of the population (91.3%) lives in private housing [40]. Even young people strive to acquire housing, so a lot of attention is paid to the quality of housing and living conditions. For people with a high standard of living and who care about the environment, environmental stressors are a very strong irritant. In contrast, lower socio-economic class individuals may experience environmental adversities more frequently and may perceive mold exposure as one of many chronic stressors rather than a salient or exceptional threat [41]. These scientific publications confirm that the same environmental stressor (mold) can have a stronger psychological impact on individuals of a higher socioeconomic class because it violates their expectations of control and status and activates important psychosocial stress pathways [20,38,39]. This claim is supported by our study’s data that physically active individuals who are more concerned about a healthy lifestyle experience more stress due to the quality of the indoor environment of the home. Summing up the discussion, the home environment is an important factor that could be associated with the development of depressive symptoms. Very often, this factor is not evaluated by either public health or mental health professionals. When assessing and treating depressive disorders, psychiatrists should treat the person, not the illness. This implies a holistic approach to the biopsychosocial and environmental system. Indoor environmental quality, particularly chronic moisture intrusion and mold contamination, is an under-recognized but modifiable factor that may contribute to effective treatment of depression.

### Strengths and Limitations

This study has several limitations that should be considered when interpreting the findings. First, the cross-sectional design of our study limits the interpretation of findings, as it does not allow for the evaluation of causal relationships between indoor environmental factors and depression symptoms. Second, most environmental factors were assessed using self-reported questionnaires without quantitative measurement, which may be subject to recall or subjective sensitivity bias, particularly when assessing odors or the presence of mold. This suggests that the actual mechanisms may be more complex, involving both psychosocial and physiological pathways. However, according to Bluyssen (2016), subjective indicators are essential for understanding real occupant discomfort, as physical measurements alone cannot fully explain how people experience the indoor environment [29]. Third limitation—some confounders appear to be missing in the current model specification. Factors such as chronic diseases, medication use, sleep quality, housing characteristics, and time spent indoors may influence both exposure and outcomes, yet they were not available in our study. Fourth limitation—the possibility of common-method bias, which cannot be excluded. Common-method bias occurs when both the exposure and the outcome are measured using the same method, typically self-reported questionnaires completed at the same time. Because the same respondent provides all information, their answers may be influenced by shared perceptions, expectations, or mood.

Despite these limitations, the study has several important strengths. A key advantage is the use of a randomly selected population-based sample, which enhances the representativeness of the findings and reduces the likelihood of selection bias. This study has a holistic approach to risk factors for depression, integrating environmental, socio-economic, and lifestyle factors. Particular attention is paid to subjective assessments of the living environment (poor indoor air quality, odors, mold, and indoor ventilation), linking them to some socio-demographic and lifestyle factors, which, according to our data, can strongly modulate the development of depressive symptoms. The observation that individuals with higher socioeconomic status and greater health awareness are more sensitive to environmental stressors suggests that the risk of depression is not directly proportional to social status; rather, a mismatch between expectations and environmental conditions may have a greater psychological impact. Future research should seek to confirm the discussion about the interaction between environment and mental health and highlight the underlying mechanisms that deserve further investigation.

## 5. Conclusions

This study demonstrated that depressive symptoms among adults are associated with a combination of sociodemographic, lifestyle, and indoor environmental factors. Female sex, younger age, single or non-partnered marital status, low physical activity, and poorer self-rated economic situation were all associated with a higher likelihood of depressive symptoms. Indoor environmental quality emerged as an important determinant of mental health. Poor microclimate conditions, unpleasant household odors, mold exposure, and insufficient room ventilation were associated with depressive symptoms. The association between ventilation and depressive symptoms differed by family status. These findings highlight that indoor environmental conditions play a meaningful role in mental well-being and are not uniform across different segments of the population. Targeted public health strategies aimed at improving indoor environmental quality and addressing vulnerabilities in specific sociodemographic groups may help reduce the burden of depressive symptoms and promote better mental health outcomes.

## Figures and Tables

**Table 1:** Baseline characteristics of responders of the Kaunas health survey (2023–2024).

Variables	Value
Number of responders, N	3175
Age, years, mean (SD)	49.4 (11.12)
Male, %	43.6
Education, %	
Secondary	31.4
College	18.8
University	49.8
Marital status, %	
Single + Divorced + Widowed	27.2
Married + Cohabiting	72.8
Body mass index, kg/m^2^, mean (SD)	27.3 (5.27)
Body mass index groups %	
Normal	36.2
Overweight	37.6
Obesity	26.2
Regular smokers, %	19.5
Physical activity in leisure time, hours/week, median	7
Depressive symptoms (CES-D-10 ≥ 4), %	18.6
The family’s economic situation, %	
Very good	67.3
Good	26.8
Poor	5.9
Member of a club/organization, %	21.2
Indoor environmental factors	
Microclimate, median	7
Odors, median	6
Mold, %	11.4
Room ventilation daily, %	94.1

**Table 2:** Distribution of responders of the Kaunas health survey (2023–2024) with depressive symptoms according to baseline characteristics.

Characteristic		Depressive	Symptoms % (n)	*p*
		No	Yes	
Sex	Males	84.5 (1170)	15.5 (214)	
	Females	79.0 (1415)	21.0 (376) ^a^	<0.001
Age groups (years)	25–34	75.3 (278)	24.7 (91)	
	35–44	82.6 (637)	17.4 (134) ^a^	
	45–54	83.6 (664)	16.4 (130) ^a^	0.006
	55+	81.1 (1006)	18.9 (235)	
Education	Secondary	80.7 (805)	19.3 (193)	
	College	80.0 (477)	20.0 (119)	0.341
	University	82.4 (1302)	17.6 (278)	
Marital status	Single/Divorced/Widowed	74.0 (639)	26.0 (225)	<0.001
	Married/Cohabiting	84.2 (1943)	15.8 (365) ^a^	
Body mass index groups	Normal	78.1 (897)	21.9 (252)	
	Overweight	83.2 (992)	16.8 (201) ^a^	0.001
	Obesity	83.5 (695)	16.5 (137) ^a^	
Smoking status (males)	Never/former	85.5 (872)	14.5 (148)	0.108
	Regular	81.8 (297)	18.2 (66)	
Smoking status (females)	Never/former	79.6 (1222)	20.4 (313)	0.136
	Regular	75.4 (193)	24.6 (63)	
Physical activity	No (1 tertile)	78.3 (855)	21.7 (237)	<0.001
	Yes (2 + 3 tertile)	83.1 (1721)	16.9 (349) ^a^	
The family’s economic situation	Very good	83.7 (1788)	16.3 (349)	
	Good	80.9 (688)	19.1 (162)	<0.001
	Poor	58.0 (109)	42.0 (79) ^a,b^	
Member of a club/organization	No	80.9 (2022)	19.1 (477)	0.131
	Yes	83.5 (563)	16.5 (111)	
Microclimate	<median	85.6 (1274)	14.4 (214)	<0.001
	≥median	77.7 (1311)	22.3 (376) ^a^	
Odors	<median	85.6 (1137)	14.4 (192)	<0.001
	≥median	78.4 (1448)	21.6 (398) ^a^	
Mold	No	82.5 (2321)	17.5 (493)	<0.001
	Yes	73.1 (264)	26.9 (97) ^a^	
Room ventilation daily	Yes	81.8 (2444)	18.2 (544)	<0.021
	No	75.4 (141)	24.6 (46) ^a^	

*p* < 0.05-values were considered statistically significant for assessing the differences in categorical variables. ^a^ *p* < 0.05 Z test with Bonferroni corrections, compared with the first group; ^b^ *p* < 0.05 Z test with Bonferroni corrections, compared with the second group.

**Table 3:** Associations of sociodemographic, lifestyle factors, family’s economic situation, membership in a club/organization, and indoor environmental factors with depressive symptoms among the 25–69-year-old population.

Variables		MODEL 1			MODEL 2	
	OR	95% CI	*p*	OR	95% CI	*p*
Age groups, years						
25–34	1			1		
35–44	**0.64**	**0.48–0.87**	**0.004**	**0.66**	**0.48–0.91**	**0.010**
45–54	**0.60**	**0.44–0.81**	**<0.001**	**0.65**	**0.48–0.90**	**0.009**
55+	**0.71**	**0.54–0.94**	**0.017**	0.86	0.64–1.16	0.324
Sex (females vs. males)	**1.45**	**1.21–1.75**	**<0.001**	**1.35**	**1.10–1.65**	**0.004**
Education						
Primary + Vocational + Secondary	1			1		
College	1.04	0.81–1.34	0.760	1.05	0.82–1.38	0.721
University	0.89	0.73–1.09	0.265	0.99	0.79–1.24	0.928
Marital status (Single, Divorced, Widowed vs. Married, Cohabiting)	**1.87**	**1.55–2.26**	**<0.001**	**1.65**	**1.35–2.01**	**<0.001**
Smoking habits (smokers vs. never/former)	1.20	0.96–1.49	0.109	1.14	0.90–1.45	0.269
Body mass index						
Normal	1			1		
Overweight	**0.72**	**0.59–0.89**	**0.002**	**0.79**	**0.63–0.98**	**0.032**
Obesity	**0.70**	**0.56–0.88**	**0.003**	**0.69**	**0.54–0.88**	**0.003**
Physical activity in leisure time						
1 tertile	1			1		
2 + 3 tertile	**0.72**	**0.61–0.88**	**0.001**	**0.78**	**0.64–0.94**	**0.010**
The family’s economic situation						
Very good	1			1		
Good	1.21	0.98–1.48	0.074	1.06	0.85–1.30	0.619
Poor	**3.71**	**2.72–5.07**	**<0.001**	**3.01**	**2.16–4.20**	**<0.001**
Membership in a club/organization	0.84	0.67–1.05	0.121	0.91	0.72–1.16	0.464
Indoor environmental factors						
Microclimate (Poor vs. Good)	**1.71**	**1.42–2.05**	**<0.001**	**1.40**	**1.14–1.71**	**0.001**
Odors (Yes vs. No)	**1.63**	**1.35–1.97**	**<0.001**	**1.45**	**1.18–1.77**	**<0.001**
Mold (Yes vs. No)	**1.73**	**1.34–2.23**	**<0.001**	**1.53**	**1.17–2.01**	**0.002**
Room ventilation daily (No vs. Yes)	**1.47**	**1.04–2.07**	**<0.001**	1.26	0.87–1.82	0.216

**Model 1:** simple binary logistic regression for each independent variable, presented in Table 3. The dependent variable: depressive symptoms. **Model 2:** multivariable binary logistic regression analysis. The dependent variable was depressive symptoms. Independent variables: sex, age, education, marital status, lifestyle factors (smoking status, body mass index, physical activity status in leisure time), family’s economic situation, membership of a club/organization, living environmental factors (microclimate, odors, mold, room ventilation daily). Bold typeface indicates significance. **OR**—odds ratio adjusted for variables according to models. **CI**—confidence interval.

**Table 4:** Associations of indoor environmental factors with depressive symptoms in different sexes, ages, family’s economic situation, marital status, and physical activity responder groups.

	OR	95% CI	*p*
**Men**			
Microclimate (Poor vs. Good)	1.33	0.95–1.86	0.094
Odors (Yes vs. No)	**1.46**	**1.05–2.05**	**0.026**
Mold (Yes vs. No)	1.37	0.87–2.17	0.18
Room ventilation daily (No vs. Yes)	1.52	0.93–2.49	0.095
**Women**			
Microclimate (Poor vs. Good)	**1.46**	**1.13–1.90**	**0.003**
Odors (Yes vs. No)	**1.47**	**1.14–1.91**	**0.003**
Mold (Yes vs. No)	**1.62**	**1.16–2.25**	**0.004**
Room ventilation daily (No vs. Yes)	1.03	0.59–1.79	0.929
**Family’s economic situation is very good + good**			
Microclimate (Poor vs. Good)	**1.37**	**1.11–1.69**	**0.003**
Odors (Yes vs. No)	**1.52**	**1.22–1.87**	**<0.001**
Mold (Yes vs. No)	**1.59**	**1.19–2.09**	**0.001**
Room ventilation daily (No vs. Yes)	1.42	0.98–2.08	0.066
**Family’s economic situation is poor**			
Microclimate (Poor vs. Good)	**2.43**	**1.12–5.28**	**0.025**
Odors (Yes vs. No)	0.93	0.45–1.93	0.839
Mold (Yes vs. No)	0.94	0.40–2.23	0.890
Room ventilation daily (No vs. Yes)	0.54	0.15–1.98	0.168
**Marital status: single, divorced, widowed**			
Microclimate (Poor vs. Good)	**1.64**	**1.16–2.32**	**0.005**
Odors (Yes vs. No)	**1.43**	**1.02–2.02**	**0.040**
Mold (Yes vs. No)	1.57	0.97–2.55	0.067
Room ventilation daily (No vs. Yes)	0.64	0.34–1.21	0.168
**Marital status: married, cohabiting**			
Microclimate (Poor vs. Good)	**1.29**	**1.00–1.67**	**0.047**
Odors (Yes vs. No)	**1.50**	**1.16–1.94**	**0.002**
Mold (Yes vs. No)	**1.45**	**1.04–2.01**	**0.027**
Room ventilation daily (No vs. Yes)	**1.81**	**1.15–2.83**	**0.010**
**Age group 25–34 years**			
Microclimate (Poor vs. Good)	1.47	0.82–2.65	0.200
Odors (Yes vs. No)	**1.88**	**1.02–3.44**	**0.042**
Mold (Yes vs. No)	1.37	0.65–2.21	0.410
Room ventilation daily (No vs. Yes)	0.49	0.18–1.28	0.145
**Age group 35–44 years**			
Microclimate (Poor vs. Good)	1.41	0.91–2.18	0.120
Odors (Yes vs. No)	1.16	0.75–1.79	0.508
Mold (Yes vs. No)	**1.88**	**1.15–3.05**	**0.011**
Room ventilation daily (No vs. Yes)	1.80	0.95–3.43	0.074
**Age group 55+ years**			
Microclimate (Poor vs. Good)	**1.47**	**1.07–2.01**	**0.018**
Odors (Yes vs. No)	**1.54**	**1.12–2.11**	**0.008**
Mold (Yes vs. No)	1.60	0.97–2.64	0.066
Room ventilation daily (No vs. Yes)	1.78	0.92–3.45	0.086
**Physically inactive (1 tertile)**			
Microclimate (Poor vs. Good)	**1.56**	**1.12–2.18**	**0.008**
Odors (Yes vs. No)	**1.44**	**1.03–2.02**	**0.036**
Mold (Yes vs. No)	1.52	0.99–2.33	0.055
Room ventilation daily (No vs. Yes)	0.82	0.44–1.53	0.538
**Physically active (2 + 3 tertiles)**			
Microclimate (Poor vs. Good)	1.25	0.97–1.63	0.082
Odors (Yes vs. No)	**1.50**	**1.161.94**	**0.002**
Mold (Yes vs. No)	**1.56**	**1.10–2.21**	**0.013**
Room ventilation daily (No vs. Yes)	**1.65**	**1.1–2.62**	**0.033**

**Model: multivariable binary logistic regression analysis.** The dependent variable was depressive symptoms. Independent variables: living environmental factors (microclimate, odors, mold, room ventilation daily). Covariates: sex, age, education, marital status, lifestyle factors (smoking status, body mass index, physical activity status in leisure time), family’s economic situation, membership of a club/organization. Bold typeface indicates significance. **OR**—odds ratio adjusted for variables according to models. ** CI**—confidence interval.

## Data Availability

The original contributions presented in this study are included in the article; further inquiries can be directed to the corresponding author.

## Abbreviations

CES-D	10-Studies Depression Scale 10
CI	confidence interval
OR	odds ratio adjusted for variables according to models
SD	standard deviation
WHO MONICA	World Health Organization Multinational Monitoring of Trends and Determinants in Cardiovascular Disease
